# Dynamic Changes in Cognitive Function in Patients With Radiation-Induced Temporal Lobe Necrosis After IMRT for Nasopharyngeal Cancer

**DOI:** 10.3389/fonc.2020.00450

**Published:** 2020-04-22

**Authors:** PeiYao Liu, XiaoShuang Niu, Dan Ou, JianJian Qiu, PengRong Lou, LiangJun Xue, Xin Zhou, Tingting Xu, XiaoShen Wang

**Affiliations:** ^1^Department of Radiation Oncology, Fudan University Shanghai Cancer Center, Shanghai, China; ^2^Department of Oncology, Shanghai Medical College, Fudan University, Shanghai, China; ^3^Department of Radiation Therapy, Huadong Hospital Affiliated to Fudan University, Shanghai, China; ^4^Center of Chemoradio-Oncology, Ningbo First Hospital, Ningbo, China; ^5^Department of Radiation Oncology, Yijishan Hospital of Wannan Medical College, Wuhu, China

**Keywords:** intensity-modulated radiation therapy, temporal lobe necrosis, cognitive function, nerve growth factor, steroids

## Abstract

**Purpose:** Radiation-induced temporal lobe necrosis (TLN) was once regarded as a progressive and irreversible disease in the era of two-dimensional radiotherapy. However, in the era of intensity-modulated radiotherapy (IMRT), the long-term development process of TLN remains unknown. We performed a prospective study to evaluate the dynamic changes in cognitive function in patients with TLN after definitive IMRT for nasopharyngeal carcinoma (NPC).

**Methods:** The enrollment criteria were as follows: (1) patients must have had confirmed NPC and must have received only one course of definitive IMRT; (2) patients radiologically diagnosed with TLN during follow-up; (3) patients with TLN who had not undergone surgical resection; and (4) patients with TLN with a follow-up period of more than 2 years. Cognitive function was measured with the mini-mental state examination (MMSE) at an interval of every 3 months. Changes in the size of the necrotic mass in the temporal lobe were evaluated by magnetic resonance imaging. The treatment interventions included the wait-and-see policy or the administration of nerve growth factor (NGF) combined with pulsed steroids.

**Results:** From January 2008 to December 2017, 86 patients with TLN entered this study. With a median follow-up of 32 months (26–50 months), 60 patients (70%) showed normal cognitive function as quantified by MMSE scores (≥27). Twenty-six patients (30%) demonstrated obvious cognitive impairment (MMSE scores ≤ 26) during follow-up. However, after receiving NGF combined with pulsed steroids, cognitive function improved significantly, and 21 of 26 patients demonstrated recovery to normal levels. Magnetic resonance imaging studies demonstrated that 10 patients had a complete response (CR), 13 had a partial response, and 3 had stable disease.

**Conclusions:** In the IMRT era, TLN is not always a progressive disease. Most patients remain stable both in their cognitive function and in the size of the necrotic mass. For patients with progressive TLN, active intervention with the administration of NGF and pulsed steroids not only can improve cognitive function but also can decrease the size of the necrotic mass.

## Introduction

Definitive radiotherapy (RT) is the mainstream treatment modality for nasopharyngeal carcinoma (NPC). Because of its specific anatomy and biologic behavior, the temporal lobe near the skull base is impossible to spare using conventional RT techniques (1). Radiation-induced temporal lobe necrosis (TLN) was a common late complication in the era of two-dimensional (2D) RT, the reported incidence ranged from 4.6% in 10 years (2 Gy per fraction to a total dose of 66 Gy) to 35% in 3.5 years (total dose 71.2 Gy with accelerated hyperfractionation) (2, 3). In patients who have received reirradiation for local recurrence, the incidence is even higher. According to Leung et al.'s (4, 5) study, the 5-year central nervous system complication-free rate was unsatisfactory, ranging from 47.8 to 55.7%.

Temporal lobe necrosis may cause cognitive impairment, mild dizziness, personality changes, headache, or general seizures (6). These symptoms severely affect a patient's quality of life (QoL). Because the typical manifestation of TLN on computed tomography or magnetic resonance imaging (MRI) is a necrotic mass surrounded with a large area of edema, a common practice for treating TLN is to control edema-related symptoms with large-dosage corticosteroids. Antiplatelet agents, high-dose vitamins, hyperbaric oxygen, and debulking resection of the necrotic mass have also been tried as treatments for this condition (7–10). However, none of these approaches have been proved to be effective for reversing cerebral necrosis up to date. Therefore, radiation-induced brain necrosis was regarded as one of the most serious complications of RT in the past decades, usually presented with a progressive and irreversible course (11).

With advances in treatment planning systems, especially the application of intensity-modulated RT (IMRT), it is possible to spare large portions of the temporal lobe from receiving high doses of radiation in patients with NPC. Consequently, both the incidence and severity of TLN may be reduced or even avoided with new techniques. In a retrospective study (12) comparing the incidence of TLN in NPC patients treated with IMRT or two-dimensional conventional RT (2D-RT), 500 patients completed at least 6 months of follow-up after RT. The results demonstrated that the IMRT technique significantly decreased the incidence of TLN, and the actuarial incidence of TLN at 5 years was 16.0% in the IMRT group and 34.9% in the 2D-RT group (*p* < 0.001). However, the incidence of TLN remained high in T4 disease regardless of the RT technique (52.6% in the IMRT group and 67.4% in the 2D-RT group, *p* = 0.680). However, cognitive function in patients with TLN was not mentioned in this report. To date, a series of studies have explored QoL, including cognitive outcomes, in IMRT-treated NPC survivors (13–16). However, there is still a lack of data on cognitive function in long-term survivors with TLN who have been treated with IMRT. Would their cognitive function remain stable or deteriorate with time? Was there any effective intervention that improved cognitive deficiency? These questions remain puzzling to most researchers. Therefore, we performed a prospective study to evaluate the dynamic changes in cognitive function in patients with TLN after definitive IMRT for NPC.

## Methods and Materials

### Patient Selection

Patients had to have undergone definitive IMRT with or without chemotherapy for histologically confirmed NPC years before: 2.0–2.2 Gy per fraction with five daily fractions per week, for a total dose of 66–70.4 Gy. They were required to undergo routine follow-up with MRI every 3–6 months. Once a patient was radiologically diagnosed with TLN, both his/her cognitive function and the size of the necrotic mass were evaluated every 3 months. To explore the dynamic changes in cognitive function and the size of the necrotic mass in the long term, only patients with a minimum follow-up time of 24 months were entered in the final analysis. However, patients with TLN resulting from the second course of IMRT for recurrent NPC were not included in this study. In addition, TLN patients combined with either of the following situation were excluded from this study: TLN together with local or regional recurrence, or TLN combined with distant metastasis, or TLN with a second primary malignancy, or debulking surgery of the necrotic mass.

### Evaluation of Cognitive Function

Once a patient was diagnosed with TLN, his/her cognitive function was evaluated with the Mini-Mental State Examination (MMSE) (17) every 3 months. Cognitive function was classified into the following four levels: normal, with a total MMSE score between 27 and 30; mild dysfunction, with a total MMSE score between 21 and 26; moderate dysfunction, with an MMSE score between 10 and 20; and severe dysfunction, with an MMSE score between 0 and 9. Dynamic changes in the MMSE score were recorded at each follow-up.

### Evaluation of the Necrotic Mass

To explore the dynamic change in the size of the necrotic mass, we explored the same procedures as described in our previous study (18). The MRI studies for TLN contained pre- and post-gadolinium administration sequences. The scans acquired before the administration of gadolinium were sagittal T1-weighted images (4 mm sections, contiguous) and axial T1- and T2-weighted images (6 mm sections, 1 mm gap). The images obtained after the administration of gadolinium were as follows: T1-weighted images (6-mm sections, 1-mm gap) and axial and coronal thin-section images (4-mm sections, contiguous) [fast-spoiled gradient recalled echo (FSPGR) acquisition, repetition time 120–215 ms, time to echo 2.2–2.8 ms]. All MRI studies were performed on a 1.5-T MRI machine (GE Healthcare, Waukesha, WI, USA) by the same team of medical imaging technologists. And all the scanned pictures were assessed by the same neuroradiologists. The size of the necrotic mass was measured in two dimensions on T1-weighted images after contrast enhancement. The prognosis of TLN was measured by the Response Evaluation Criteria in Solid Tumors (RECIST) (19).

### Treatment Intervention

Treatment was prescribed at the discretion of the physicians. For patients with normal cognitive function, the “wait and see” policy was adopted. Patients with cognitive deficits received nerve growth factor (NGF) combined with pulsed corticosteroids. Nerve growth factor was dissolved in 2 mL normal saline and then injected intramuscularly once a day at 18 μg/time, with a continuous use for 2 months (18).

### Statistical Analysis

Variables that were potentially associated with cognitive function, such as age, gender, size of the necrotic mass, maximal radiation dose to the normal brain, and the interval between radiation and confirmation of necrosis, were analyzed by independent-samples test.

## Results

### Characteristics of the Enrolled Patients

From January 2008 to December 2017, 95 patients were radiologically diagnosed with TLN, but the routine follow-up time was <12 months in nine cases. The remaining 86 cases were included in this study.

Sixty-two patients were male, and 24 were female. The median age at diagnosis was 53.6 years old. The mean latency between the diagnosis of TLN and primary IMRT was 26.7 months (from 14 to 64 months). The median follow-up time was 32 months (26–50 months). Seventy-five patients (87.2%) had unilateral TLN, and 11 (12.8%) had bilateral TLN. The vast majority (84 of 86) had T3–T4 primary disease, and only two patients had T2 primary disease according to the TNM staging system (AJCC eighth edition). All patients received IMRT combined with chemotherapy. The detailed treatment modalities were induction chemotherapy followed by concurrent chemo-IMRT in 51 patients, concurrent chemo-IMRT followed by adjuvant chemotherapy in 29 patients, and concurrent chemo-IMRT in 6 patients. Details of the cohort are shown in Table 1. Concurrent chemotherapy regimen was cisplatin alone at 80 mg/m^2^ on day 1, repeated every 3 weeks, and the induction or adjuvant chemotherapy regimen was docetaxel (75 mg/m^2^, day 1) and cisplatin (75 mg/m^2^, Day 1) with or without 5-Fu [500 mg/m^2^, continuous intravenous infusion (CIV) for 120 h], repeated every 21 days.

**Table 1:** Characteristics of the 86 enrolled patients with TLN.

Sex	
Male	62
Female	24
Median age, years	53.6 (range, 32–75)
Mean latency, months	26.7 (range, 14–64)
Median follow-up, months	32 (range, 26–50)
Location	
Unilateral	76
Bilateral	10
Maximum dose of the necrotic area	68.2–74.3 Gy
T stage at presentation	
T2	2
T3	11
T4	73
Previous treatment modality	
Concurrent chemo-RT	6
IC+concurrent chemo-RT	51
Concurrent chemo-RT + AC	29
Cognitive function upon TLN diagnosis	
Normal	75
Mild impairment	11
Moderate dysfunction	0
Severe impairment	0
Maximum diameter upon TLN diagnosis	
≤ 2 cm	68
2-4 cm	15
≥4 cm	3
Intervention for TLN	
Wait and see	60
NGF plus steroids	26

*IC, induction chemotherapy; AC, adjuvant chemotherapy; TLN, temporal lobe necrosis; NGF, nerve growth factor*.

### Dynamic Change in Cognitive Function

Upon diagnosis of TLN, 75 of 86 patients demonstrated normal cognitive function as quantified by an MMSE score ≥27. Eleven patients had mild cognitive deficiency, and their MMSE scores ranged between 21 and 26. No patients were found to have MMSE scores ≤ 20. With a median follow-up of 32 months (26–50 months), 70% (60 of 86) of patients had stable cognitive function, and their MMSE scores remained between 27 and 30 at each follow-up. Although no treatment intervention was prescribed to them, their cognitive function was maintained at a normal level, which lasted for more than 2 years. Fifteen patients had progressive disease (PD) during follow-up, and their cognitive function declined from normal to mild impairment. Altogether, 26 patients (18 males and 8 females), revealed obvious cognitive impairment (MMSE scores ranged between 21 and 26), and they received NGF combined with pulsed steroids. After the treatment intervention, 21 of 26 patients (80.8%) recovered to normal cognitive function, as indicated by the MMSE scores (≥27). Only five patients showed no response to treatment, but their cognitive function did not deteriorate after intervention, which means that no patients were found to have moderate or severe cognitive impairment during the long-term follow-up.

### Dynamic Change in the Necrotic Mass

**Figure 1:**
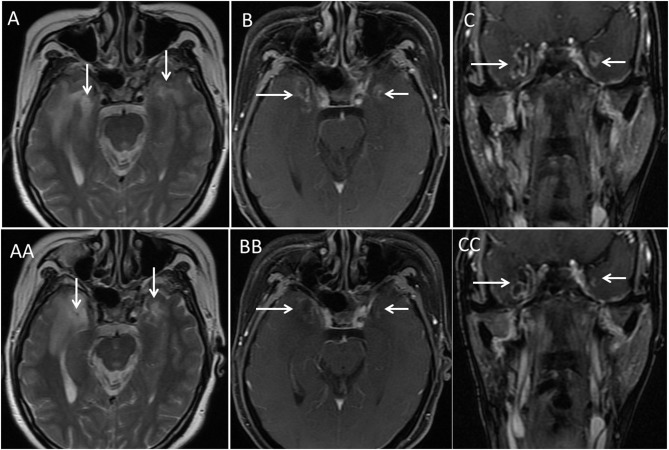
Illustration of bilateral TLN (arrows) on MRI. This was a 47-year-old woman with T4N1 nasopharyngeal cancer. She received induction chemotherapy followed by concurrent chemo-IMRT. Routine follow-up at 61 months after IMRT demonstrated bilateral TLN (arrows) on axial T2-weighted MRI **(A)** and postcontrast axial **(B)** and coronal **(C)** T1-weighted MRI. However, she had normal cognitive function (MMSE score 28). She underwent the wait-and-see policy, and no intervention was prescribed to her. With long-term follow-up (28 months), she did not show impaired cognitive function. The necrotic mass remained nearly unchanged on axial T2-weighted MRI **(AA)** and postcontrast axial **(BB)** and coronal **(CC)** T1-weighted MRI.

**Figure 2:**
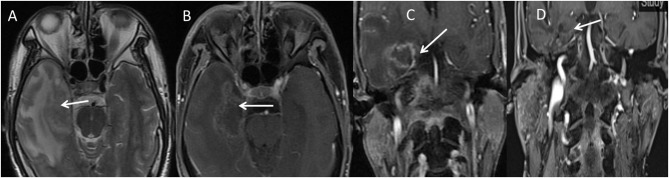
Illustration of typical TLN necrosis (arrow) on MRI images. This was a 49-year-old male patient. When diagnosed with TLN, he had mild cognitive impairment (MMSE score 23). However, after treatment with nerve growth factor and pulsed steroids, his cognitive function returned to normal (MMSE score 28), and the necrotic mass completely disappeared. **(A)** Necrotic mass (arrow) in the right temporal lobe surrounded by a large area of edema on axial T2-weighted MRI. **(B)** Necrotic mass (arrow) in the right temporal lobe on axial contrast-enhanced T1-weighted MRI with fat suppression. **(C)** Necrotic mass (arrow) in the right temporal lobe on coronal contrast-enhanced T1-weighted MRI with fat suppression. **(D)** After intervention with nerve growth factor and pulsed steroids, the necrotic mass in the right temporal lobe completely disappeared, as shown on coronal contrast-enhanced T1-weighted MRI with fat suppression, and his cognitive function recovered to a normal level (MMSE score 29).

When first diagnosed with TLN, 76 patients had bilateral disease, and 10 had unilateral disease. The size of the necrotic mass was measured on T1-weighted MRI after contrast enhancement. A total of 79% (68 of 86) of patients had a necrotic mass ≤ 2 cm in maximum diameter; 17.4% (15 of 86) of the patients' necrotic masses ranged from 2 to 4 cm in maximum diameter. Only three patients had necrotic disease ≥4 cm in maximum diameter at presentation. During more than 2 years of follow-up, the size of the necrotic mass remained stable disease (SD) in 53 patients according to the RECIST criteria (Figure 1). Seven patients did not receive any treatment, but their necrotic mass spontaneously disappeared [complete response (CR)] on MRI. Clinical characteristics of the seven patients were listed in Table 2. Twenty-six patients had PD and received NGF combined with pulsed steroids. After the treatment intervention, RECIST revealed that 10 patients achieved a CR (Figure 2), 13 achieved a partial response (PR), and 3 achieved SD. Altogether, the necrotic mass completely disappeared in 17 patients; the average time intervals to TLN improvement in the 7 spontaneous remissions and 10 remissions after treatment intervention were 22 and 18.5 months, respectively (independent-samples test, *p* = 0.782).

**Table 2:** Details of the 7 patients with spontaneous remissions of TLN.

Sex	
Male	5
Female	2
Median age, years	51.4 (range, 34–70)
Mean latency, months	26.7 (range, 18–56)
Median follow-up, months	30 (range, 24–46)
Location	
Unilateral	6
Bilateral	1
Maximum dose of the necrotic area	68.4–73.8 Gy
T stage at presentation	
T3	4
T4	3
Previous treatment modality	
IC+concurrent chemo-RT	5
Concurrent chemo-RT + AC	2
Cognitive function upon TLN diagnosis	
Normal	7
Mild impairment	0
Maximum diameter upon TLN diagnosis	
≤ 2 cm	7
2-4 cm	0

*IC, induction chemotherapy; AC, adjuvant chemotherapy; TLN, temporal lobe necrosis*.

### Correlation Between Cognitive Impairment and the Size of the Necrotic Mass

Of the 75 patients who had normal cognitive function upon the diagnosis of TLN, 68 patients had necrotic masses ≤ 2 cm in maximum diameter, and 7 had necrotic masses ranging between 2.1 and 2.5 cm in maximum diameter. The size of the necrotic mass in the 11 patients with mild cognitive impairment was ≥3.0 cm in maximum diameter (independent-samples test, *p* = 0.016). During follow-up, 60 patients maintained normal cognitive function, and all were found to have necrotic masses <2 cm in maximum diameter as measured on MRI. Of the 26 patients who had mild cognitive impairment, all had necrotic masses larger than 3.5 cm in maximum diameter before treatment (independent-samples test, *p* = 0.008). After the treatment intervention with NGF plus pulsed steroids, 21 of 26 patients (80.8%) recovered to normal cognitive function. The MRI study revealed that 10 patients' necrotic masses completely disappeared, and 11 patients' necrotic masses shrank to <2 cm in maximum diameter. Five patients remained mild cognitive dysfunction despite the treatment intervention, but all their necrotic masses were larger than 3 cm. Taken all the information pre-intervention and post-intervention, we infer that 2 cm in maximum diameter might be a cutoff value for predicting cognitive impairment (independent-samples test, *p* = 0.022).

### Other Factors That Might Be Related to the Development of TLN

Twenty-six patients had cognitive impairment during follow-up, but before treatment intervention, gender distribution was 18 of 62 males and 8 of 24 females (independent-samples test, *p* = 0.697). The mean age of the 26 patients was 53.1 years old, whereas the mean age of patients with SD was 56.2 years (*p* = 0.249). The time intervals between RT and diagnosis of TLN in patients with stable and declined cognitive function were 40.3 and 37.5 months, respectively (*p* = 0.673). The mean maximum doses of TLN in the 26 patients with PD and in the 60 patients with SD were 71.2 and 70.6 Gy, respectively (*p* = 0.486). In the end, the necrotic mass in 17 patients completely disappeared (7 spontaneous remissions and 10 remissions after treatment intervention). There were 11 males and 6 females (*p* = 0.448). The mean maximum doses of TLN in the 17 patients with CR and in the 69 patients with PR or SD were 71.0 and 70.7 Gy, respectively (independent-samples test, *p* = 0.577). The mean ages of the 17 and 69 patients were 58.1 and 56.2 years, respectively (independent-samples test, *p* = 0.27).

### Adverse Events of Treatment for TLN

Of the 26 patients who received NGF combined with pulsed steroids, no severe adverse events were found, except that 13 patients complained of soreness at the injection site. Of the 86 patients with TLN, 12 patients died of multiple metastases during long-term follow-up, but there were no TLN-related deaths.

## Discussion

Radiotherapy for NPC is associated with some inevitable acute and late complications, such as mucositis, dermatitis, xerostomia, hearing loss, skin fibrosis, and brain injury. Radiotherapy is the only radical cure in non-metastatic NPC. Radiotherapy alone is adequate for early-stage NPC, whereas RT combined with chemotherapy is required for locally advanced disease. Because the temporal lobes are located just above the skull base and because of the need for adequate coverage of all potential sites of invasion, the lower part of the bilateral temporal lobes is unavoidably included in the radiation field using conventional RT technique (1). Therefore, TLN seems to be inevitable in the era of 2D-RT. However, IMRT technique can reduce both radiation dose and the volume of the irradiated temporal lobes. Thus, both the incidence and severity of TLN might be decreased. A series of clinical studies have proved this viewpoint. A recent research by Zhou et al. (12) analyzed 195 NPC patients who received 2D-RT and 305 patients treated with IMRT. The results demonstrated that the incidence of TLN was 16.0% in the IMRT group and 34.9% in the 2D-RT group at 5 years. The advanced IMRT technique significantly decreased the incidence of TLN (*p* < 0.001). The authors mentioned that not all types of TLN negatively impacted the patient's QoL. This study showed that part of the TLN patients had normal neurocognitive function; only focal necrosis was manifested on routine MRI follow-up; both the size of necrosis and the patent's QoL remained stable in the long term. However, the exact proportion was not given in this report. Lam et al. (20) retrieved a large number of patients with primary or recurrent NPC treated in Tuen Mun Hospital from January 1990 to October 2008. The RT techniques included 2D-RT, 3D-CRT, IMRT, and brachytherapy. Among the 174 cases of radiologically confirmed TLN, 79 (45.3%) were without symptoms. The authors considered that asymptomatic TLN could adopt wait-and-see policy without any treatment. For patients with symptoms affecting their daily activities, treatment intervention with intermittent pulsed steroids demonstrated better clinical responses. However, this treatment had no impact on the complication-free survival rate. In our research, all patients were treated with one course of definitive IMRT, 70% (60 of 86) were asymptomatic, and they did not receive any treatment intervention. However, their cognitive function remained stable during long-term follow-up.

Although there are many tools used for neurocognitive testing, the MMSE was applied in our study because it could be quickly implemented to patients in less than 15 min and does not need special materials or testing kits that might burden providers (21). In addition, the MMSE is regarded as the “gold standard” screening instrument for global cognition (22). In recent decades, the treatment of CRN with clinical symptoms has included corticosteroids, hyperbaric oxygen, anticoagulants, high-dose vitamins, or surgery (7–10). Although symptomatic alleviation can be obtained, it is usually transient in most patients. In the past few years, some researchers have tried to use bevacizumab as the intervention modality for CRN. A prospective placebo-controlled study (23) demonstrated that both radiologic responses and symptomatic improvements could be observed after intervention with bevacizumab, whereas no patient responded to placebo. In their study, among the 11 patients who received bevacizumab, 6 had adverse events: one aspiration pneumonia, one superior sagittal sinus thrombosis, one pulmonary embolus secondary to deep vein thrombosis, and three ischemic changes. The standard treatment for symptomatic TLN is the administration of NGF combined with pulsed steroids in our hospital. Our study revealed that this practice was effective and safe. Of the 26 patients who received NGF combined with pulsed steroids, 10 achieved a CR, 13 achieved a PR, and 3 had SD. The only observed adverse event was mild pain at the injection site in 13 patients. There were no cases of treatment-related death in the 26 patients with progressive TLN.

The present study is, to our knowledge, the only one that has explored both dynamic changes in cognitive function and the size of the necrotic mass in TLN to date. Upon diagnosis of TLN, 75 of 86 patients demonstrated normal cognitive function, and 68 patients had necrotic masses ≤ 2 cm in maximum diameter. Nine patients had necrotic masses ranging from 2.1 to 2.5 cm. The size of the necrotic mass in the 11 patients with mild cognitive impairment was ≥3.0 cm in maximum diameter. During follow-up, 60 patients maintained normal cognitive function, and all were found to have necrotic masses less than 2 cm in maximum diameter as measured on MRI. Of the 26 patients who had mild cognitive impairment, all had necrotic masses larger than 3.5 cm in maximum diameter (*p* < 0.01). After treatment intervention with NGF plus pulsed steroids, 21 of 26 patients (80.8%) recovered to normal cognitive function. The MRI study revealed that 10 patients' necrotic masses completely disappeared, and 11 patients' necrotic masses shrank to <2 cm in maximum diameter. Based on our study, it is reasonable to conclude that cognitive function correlated directly with the TLN size. The larger the necrotic mass was, the greater the potential impairment in cognitive function. When the necrotic mass was smaller than 2 cm, cognitive function tended not to be impaired in our study.

### Limitations of the Present Study

It is well-known that cognitive function is affected by many other confounding factors, such as education level, underlying disease such as diabetes and hypertension, the maximum dose of irradiated normal brain, age, follow-up time, and lifestyle (15, 24, 25). In our study, the information regarding education level and accompanying disease was not included in the original medical records, so it was impossible to explore the association between cognitive function and these variables. Perhaps due to the small sample size, the impact of gender, age, the time interval between RT and the diagnosis of TLN, and maximum radiation dose to the normal brain on the development and prognosis of TLN was found to have no clinically significant difference. Therefore, a larger sample of patients with TLN from more cancer centers is needed to explore the association between cognitive function and the above mentioned variables. In addition, the MMSE score before RT was not provided, because the latency interval between radiation and diagnosis of TLN was so long in some patients (about 5 years); the second reason was that many patients received RT in other hospitals, they would not come to our center and enroll in the current study unless they were confirmed with TLN.

## Conclusions

This is the first study exploring the dynamic changes in both cognitive function and the size of the necrotic mass in patients with TLN. In the IMRT era, TLN is not always a PD. Most patients had normal cognitive function as indicated by the MMSE scores upon TLN diagnosis. A large proportion of patients remained stable both in terms of their cognitive function and in the size of the necrotic mass during long-term follow-up, even without treatment. Cognitive function correlated directly with the TLN size. The smaller the necrotic mass was, the less impairment in cognitive function; 2 cm in maximum diameter might be a cut-off value for predicting cognitive decline. For patients with progressive TLN, intervention with NGF and pulsed steroids is an effective and safe practice that not only can improve cognitive function but also can shrink the size of the necrotic mass.

## Data Availability Statement

The datasets presented in this article are not readily available because the data forms part of an ongoing study and due to institution regulation. Requests to access the datasets should be directed to the corresponding author.

## Ethics Statement

The studies involving human participants were reviewed and approved by Ethical Committee and Institutional Review Board of Fudan University Shanghai Cancer Centre (FDUSCC). The patients/participants provided their written informed consent to participate in this study. Written informed consent was obtained from the individual(s) for the publication of any potentially identifiable images or data included in this article.

## Author Contributions

PLi, XN, DO, and XW are responsible for the conception and design of the study. XN and DO collected and assembled the data. PLo did data analysis and interpretation. XW provided administrative support. All authors contributed to manuscript writing and approved the final manuscript.

## Conflict of Interest

The authors declare that the research was conducted in the absence of any commercial or financial relationships that could be construed as a potential conflict of interest.
